# Supplementary material for: Modeling glioblastoma heterogeneity as a dynamic network of cell states
Source: Mol Syst Biol. 2021 Sep 16;17(9):e10105. doi: 10.15252/msb.202010105 (PMC8444284; doi:10.15252/msb.202010105)
Supplement: Supplementary file 5 — Source Data for Figure 3 [file MSB-17-e10105-s001.zip › Figure3A_sourcedata/GSEA_3065/hallmarks_state2.GseaPreranked.1623416269049/HALLMARK_G2M_CHECKPOINT.html]

Details for gene set HALLMARK\_G2M\_CHECKPOINT[GSEA]

|  || Dataset | state2 |
| Phenotype | NoPhenotypeAvailable |
| Upregulated in class | na\_pos |
| GeneSet | HALLMARK\_G2M\_CHECKPOINT |
| Enrichment Score (ES) | 0.79641306 |
| Normalized Enrichment Score (NES) | 2.8468463 |
| Nominal p-value | 0.0 |
| FDR q-value | 0.0 |
| FWER p-Value | 0.0 |
Table: GSEA Results Summary

  

Fig 1: Enrichment plot: HALLMARK\_G2M\_CHECKPOINT      
 Profile of the Running ES Score & Positions of GeneSet Members on the Rank Ordered List

  

| PROBE | GENE SYMBOL | GENE\_TITLE | RANK IN GENE LIST | RANK METRIC SCORE | RUNNING ES | CORE ENRICHMENT || 1 | UBE2C |  |  | 9 | 0.704 | 0.0172 | Yes |
| 2 | MYBL2 |  |  | 14 | 0.600 | 0.0323 | Yes |
| 3 | TOP2A |  |  | 15 | 0.596 | 0.0477 | Yes |
| 4 | MKI67 |  |  | 17 | 0.590 | 0.0628 | Yes |
| 5 | CDK1 |  |  | 18 | 0.577 | 0.0777 | Yes |
| 6 | UBE2S |  |  | 19 | 0.574 | 0.0926 | Yes |
| 7 | MAD2L1 |  |  | 20 | 0.563 | 0.1071 | Yes |
| 8 | CKS1B |  |  | 23 | 0.554 | 0.1212 | Yes |
| 9 | CKS2 |  |  | 28 | 0.532 | 0.1345 | Yes |
| 10 | TPX2 |  |  | 29 | 0.531 | 0.1483 | Yes |
| 11 | STMN1 |  |  | 33 | 0.520 | 0.1614 | Yes |
| 12 | CENPF |  |  | 34 | 0.518 | 0.1748 | Yes |
| 13 | HMGN2 |  |  | 35 | 0.512 | 0.1880 | Yes |
| 14 | GINS2 |  |  | 37 | 0.506 | 0.2010 | Yes |
| 15 | SMC2 |  |  | 39 | 0.493 | 0.2136 | Yes |
| 16 | ORC6 |  |  | 40 | 0.493 | 0.2264 | Yes |
| 17 | DBF4 |  |  | 41 | 0.492 | 0.2391 | Yes |
| 18 | SMC4 |  |  | 42 | 0.489 | 0.2517 | Yes |
| 19 | TMPO |  |  | 43 | 0.489 | 0.2644 | Yes |
| 20 | PBK |  |  | 46 | 0.484 | 0.2766 | Yes |
| 21 | NUSAP1 |  |  | 47 | 0.483 | 0.2891 | Yes |
| 22 | KPNA2 |  |  | 50 | 0.472 | 0.3011 | Yes |
| 23 | MCM3 |  |  | 51 | 0.464 | 0.3131 | Yes |
| 24 | CHAF1A |  |  | 55 | 0.457 | 0.3246 | Yes |
| 25 | E2F1 |  |  | 59 | 0.446 | 0.3358 | Yes |
| 26 | BIRC5 |  |  | 61 | 0.443 | 0.3471 | Yes |
| 27 | PTTG1 |  |  | 63 | 0.439 | 0.3583 | Yes |
| 28 | FBXO5 |  |  | 67 | 0.420 | 0.3689 | Yes |
| 29 | LMNB1 |  |  | 69 | 0.417 | 0.3795 | Yes |
| 30 | EZH2 |  |  | 70 | 0.417 | 0.3903 | Yes |
| 31 | SNRPD1 |  |  | 74 | 0.408 | 0.4005 | Yes |
| 32 | MCM5 |  |  | 83 | 0.394 | 0.4099 | Yes |
| 33 | KIF4A |  |  | 84 | 0.393 | 0.4200 | Yes |
| 34 | KIF15 |  |  | 86 | 0.392 | 0.4300 | Yes |
| 35 | HNRNPD |  |  | 88 | 0.390 | 0.4400 | Yes |
| 36 | DTYMK |  |  | 94 | 0.379 | 0.4492 | Yes |
| 37 | CDC45 |  |  | 95 | 0.377 | 0.4590 | Yes |
| 38 | CDKN3 |  |  | 97 | 0.376 | 0.4686 | Yes |
| 39 | CENPE |  |  | 105 | 0.372 | 0.4774 | Yes |
| 40 | CDC6 |  |  | 106 | 0.372 | 0.4870 | Yes |
| 41 | NDC80 |  |  | 115 | 0.354 | 0.4953 | Yes |
| 42 | KIF23 |  |  | 117 | 0.351 | 0.5043 | Yes |
| 43 | CDKN2C |  |  | 123 | 0.341 | 0.5125 | Yes |
| 44 | KIF11 |  |  | 129 | 0.335 | 0.5207 | Yes |
| 45 | TACC3 |  |  | 130 | 0.334 | 0.5293 | Yes |
| 46 | CDK4 |  |  | 131 | 0.333 | 0.5379 | Yes |
| 47 | CCNA2 |  |  | 135 | 0.331 | 0.5461 | Yes |
| 48 | NCL |  |  | 146 | 0.316 | 0.5532 | Yes |
| 49 | RAD21 |  |  | 147 | 0.316 | 0.5614 | Yes |
| 50 | MCM6 |  |  | 149 | 0.311 | 0.5693 | Yes |
| 51 | KIF22 |  |  | 154 | 0.306 | 0.5768 | Yes |
| 52 | NASP |  |  | 156 | 0.306 | 0.5846 | Yes |
| 53 | AURKB |  |  | 163 | 0.303 | 0.5918 | Yes |
| 54 | SAP30 |  |  | 164 | 0.303 | 0.5996 | Yes |
| 55 | DDX39A |  |  | 183 | 0.293 | 0.6052 | Yes |
| 56 | BARD1 |  |  | 185 | 0.292 | 0.6127 | Yes |
| 57 | CCNB2 |  |  | 190 | 0.286 | 0.6196 | Yes |
| 58 | CDC20 |  |  | 193 | 0.284 | 0.6268 | Yes |
| 59 | HSPA8 |  |  | 195 | 0.283 | 0.6340 | Yes |
| 60 | HMGB3 |  |  | 200 | 0.282 | 0.6408 | Yes |
| 61 | KIF20B |  |  | 208 | 0.278 | 0.6473 | Yes |
| 62 | SRSF2 |  |  | 213 | 0.275 | 0.6539 | Yes |
| 63 | CHEK1 |  |  | 224 | 0.266 | 0.6597 | Yes |
| 64 | MCM2 |  |  | 228 | 0.262 | 0.6662 | Yes |
| 65 | JPT1 |  |  | 236 | 0.260 | 0.6721 | Yes |
| 66 | CENPA |  |  | 238 | 0.259 | 0.6787 | Yes |
| 67 | SMC1A |  |  | 249 | 0.253 | 0.6842 | Yes |
| 68 | KIF2C |  |  | 252 | 0.251 | 0.6905 | Yes |
| 69 | BUB3 |  |  | 311 | 0.228 | 0.6901 | Yes |
| 70 | RACGAP1 |  |  | 333 | 0.222 | 0.6936 | Yes |
| 71 | BUB1 |  |  | 335 | 0.222 | 0.6992 | Yes |
| 72 | KPNB1 |  |  | 350 | 0.216 | 0.7033 | Yes |
| 73 | HMMR |  |  | 358 | 0.215 | 0.7081 | Yes |
| 74 | PLK1 |  |  | 364 | 0.213 | 0.7130 | Yes |
| 75 | POLA2 |  |  | 366 | 0.212 | 0.7184 | Yes |
| 76 | RPA2 |  |  | 367 | 0.212 | 0.7239 | Yes |
| 77 | AURKA |  |  | 375 | 0.210 | 0.7286 | Yes |
| 78 | KNL1 |  |  | 379 | 0.209 | 0.7336 | Yes |
| 79 | BRCA2 |  |  | 427 | 0.193 | 0.7336 | Yes |
| 80 | TROAP |  |  | 435 | 0.193 | 0.7378 | Yes |
| 81 | NSD2 |  |  | 444 | 0.191 | 0.7419 | Yes |
| 82 | SYNCRIP |  |  | 503 | 0.180 | 0.7402 | Yes |
| 83 | CBX1 |  |  | 509 | 0.179 | 0.7443 | Yes |
| 84 | PRC1 |  |  | 514 | 0.178 | 0.7485 | Yes |
| 85 | TRA2B |  |  | 515 | 0.178 | 0.7531 | Yes |
| 86 | CDC25B |  |  | 519 | 0.178 | 0.7574 | Yes |
| 87 | CDC25A |  |  | 578 | 0.167 | 0.7554 | Yes |
| 88 | DKC1 |  |  | 592 | 0.165 | 0.7582 | Yes |
| 89 | KIF5B |  |  | 604 | 0.163 | 0.7613 | Yes |
| 90 | CASP8AP2 |  |  | 608 | 0.162 | 0.7651 | Yes |
| 91 | MARCKS |  |  | 630 | 0.159 | 0.7670 | Yes |
| 92 | EXO1 |  |  | 643 | 0.157 | 0.7697 | Yes |
| 93 | SRSF10 |  |  | 664 | 0.155 | 0.7716 | Yes |
| 94 | PRIM2 |  |  | 667 | 0.154 | 0.7753 | Yes |
| 95 | MAP3K20 |  |  | 678 | 0.152 | 0.7782 | Yes |
| 96 | NUP50 |  |  | 685 | 0.152 | 0.7815 | Yes |
| 97 | CTCF |  |  | 691 | 0.151 | 0.7848 | Yes |
| 98 | RAD54L |  |  | 729 | 0.146 | 0.7846 | Yes |
| 99 | MT2A |  |  | 731 | 0.146 | 0.7883 | Yes |
| 100 | NEK2 |  |  | 805 | 0.138 | 0.7839 | Yes |
| 101 | KMT5A |  |  | 821 | 0.136 | 0.7858 | Yes |
| 102 | TTK |  |  | 831 | 0.135 | 0.7883 | Yes |
| 103 | CCND1 |  |  | 840 | 0.134 | 0.7909 | Yes |
| 104 | TFDP1 |  |  | 874 | 0.130 | 0.7907 | Yes |
| 105 | CCNF |  |  | 895 | 0.128 | 0.7919 | Yes |
| 106 | POLQ |  |  | 939 | 0.125 | 0.7905 | Yes |
| 107 | SFPQ |  |  | 967 | 0.122 | 0.7907 | Yes |
| 108 | RAD23B |  |  | 973 | 0.122 | 0.7933 | Yes |
| 109 | PLK4 |  |  | 986 | 0.121 | 0.7952 | Yes |
| 110 | ILF3 |  |  | 1004 | 0.120 | 0.7964 | Yes |
| 111 | NOLC1 |  |  | 1104 | 0.111 | 0.7886 | No |
| 112 | TRAIP |  |  | 1314 | 0.097 | 0.7685 | No |
| 113 | SRSF1 |  |  | 1315 | 0.097 | 0.7710 | No |
| 114 | CDC27 |  |  | 1319 | 0.097 | 0.7732 | No |
| 115 | POLE |  |  | 1368 | 0.094 | 0.7704 | No |
| 116 | HNRNPU |  |  | 1369 | 0.094 | 0.7729 | No |
| 117 | GSPT1 |  |  | 1377 | 0.093 | 0.7745 | No |
| 118 | ODF2 |  |  | 1512 | 0.086 | 0.7622 | No |
| 119 | SMARCC1 |  |  | 1516 | 0.086 | 0.7641 | No |
| 120 | G3BP1 |  |  | 1566 | 0.083 | 0.7610 | No |
| 121 | RBL1 |  |  | 1569 | 0.083 | 0.7629 | No |
| 122 | KATNA1 |  |  | 1711 | 0.076 | 0.7496 | No |
| 123 | STIL |  |  | 1784 | 0.073 | 0.7437 | No |
| 124 | INCENP |  |  | 1833 | 0.071 | 0.7404 | No |
| 125 | CDC7 |  |  | 1846 | 0.070 | 0.7409 | No |
| 126 | XPO1 |  |  | 1871 | 0.070 | 0.7401 | No |
| 127 | ODC1 |  |  | 1913 | 0.068 | 0.7374 | No |
| 128 | HUS1 |  |  | 2021 | 0.064 | 0.7275 | No |
| 129 | UCK2 |  |  | 2073 | 0.062 | 0.7236 | No |
| 130 | E2F2 |  |  | 2176 | 0.058 | 0.7141 | No |
| 131 | HIF1A |  |  | 2182 | 0.058 | 0.7150 | No |
| 132 | PRMT5 |  |  | 2240 | 0.056 | 0.7103 | No |
| 133 | CUL3 |  |  | 2241 | 0.056 | 0.7117 | No |
| 134 | EWSR1 |  |  | 2352 | 0.052 | 0.7012 | No |
| 135 | RBM14 |  |  | 2369 | 0.052 | 0.7008 | No |
| 136 | E2F3 |  |  | 2386 | 0.051 | 0.7004 | No |
| 137 | FANCC |  |  | 2472 | 0.049 | 0.6924 | No |
| 138 | STAG1 |  |  | 2478 | 0.048 | 0.6932 | No |
| 139 | CUL1 |  |  | 2498 | 0.048 | 0.6923 | No |
| 140 | PDS5B |  |  | 2565 | 0.046 | 0.6864 | No |
| 141 | SUV39H1 |  |  | 2604 | 0.044 | 0.6834 | No |
| 142 | CHMP1A |  |  | 2609 | 0.044 | 0.6841 | No |
| 143 | YTHDC1 |  |  | 2727 | 0.041 | 0.6725 | No |
| 144 | TOP1 |  |  | 2824 | 0.038 | 0.6631 | No |
| 145 | ESPL1 |  |  | 2838 | 0.038 | 0.6627 | No |
| 146 | DR1 |  |  | 2884 | 0.037 | 0.6588 | No |
| 147 | NUP98 |  |  | 2986 | 0.034 | 0.6488 | No |
| 148 | SLC7A1 |  |  | 3014 | 0.033 | 0.6467 | No |
| 149 | CUL4A |  |  | 3030 | 0.033 | 0.6460 | No |
| 150 | WRN |  |  | 3435 | 0.025 | 0.6029 | No |
| 151 | AMD1 |  |  | 3505 | 0.024 | 0.5961 | No |
| 152 | ATF5 |  |  | 3510 | 0.024 | 0.5963 | No |
| 153 | SLC38A1 |  |  | 3562 | 0.023 | 0.5913 | No |
| 154 | ORC5 |  |  | 3711 | 0.020 | 0.5759 | No |
| 155 | HIRA |  |  | 3760 | 0.019 | 0.5712 | No |
| 156 | MYC |  |  | 3842 | 0.018 | 0.5629 | No |
| 157 | RPS6KA5 |  |  | 3910 | 0.017 | 0.5561 | No |
| 158 | CDKN1B |  |  | 4019 | 0.015 | 0.5448 | No |
| 159 | PAFAH1B1 |  |  | 4126 | 0.014 | 0.5337 | No |
| 160 | HMGA1 |  |  | 4135 | 0.013 | 0.5332 | No |
| 161 | E2F4 |  |  | 4376 | 0.009 | 0.5075 | No |
| 162 | TENT4A |  |  | 4548 | 0.007 | 0.4891 | No |
| 163 | SMAD3 |  |  | 4579 | 0.006 | 0.4861 | No |
| 164 | MNAT1 |  |  | 4598 | 0.006 | 0.4843 | No |
| 165 | SS18 |  |  | 4600 | 0.006 | 0.4843 | No |
| 166 | PURA |  |  | 5366 | -0.006 | 0.4018 | No |
| 167 | UPF1 |  |  | 5545 | -0.009 | 0.3827 | No |
| 168 | MTF2 |  |  | 5648 | -0.011 | 0.3720 | No |
| 169 | LIG3 |  |  | 5841 | -0.014 | 0.3516 | No |
| 170 | ABL1 |  |  | 5952 | -0.016 | 0.3401 | No |
| 171 | CUL5 |  |  | 6193 | -0.020 | 0.3147 | No |
| 172 | MAPK14 |  |  | 6426 | -0.024 | 0.2902 | No |
| 173 | CCNT1 |  |  | 6472 | -0.025 | 0.2860 | No |
| 174 | TNPO2 |  |  | 6518 | -0.026 | 0.2818 | No |
| 175 | FOXN3 |  |  | 6582 | -0.027 | 0.2757 | No |
| 176 | PRPF4B |  |  | 6598 | -0.027 | 0.2748 | No |
| 177 | ARID4A |  |  | 6708 | -0.030 | 0.2637 | No |
| 178 | LBR |  |  | 7064 | -0.038 | 0.2263 | No |
| 179 | ATRX |  |  | 7408 | -0.047 | 0.1905 | No |
| 180 | PML |  |  | 7505 | -0.051 | 0.1814 | No |
| 181 | NUMA1 |  |  | 7579 | -0.053 | 0.1749 | No |
| 182 | TLE3 |  |  | 7678 | -0.056 | 0.1657 | No |
| 183 | MEIS1 |  |  | 7785 | -0.059 | 0.1558 | No |
| 184 | DMD |  |  | 7987 | -0.067 | 0.1358 | No |
| 185 | SLC7A5 |  |  | 8067 | -0.070 | 0.1291 | No |
| 186 | RASAL2 |  |  | 8072 | -0.070 | 0.1304 | No |
| 187 | NOTCH2 |  |  | 8358 | -0.086 | 0.1018 | No |
| 188 | TGFB1 |  |  | 8497 | -0.095 | 0.0894 | No |
| 189 | MEIS2 |  |  | 8893 | -0.134 | 0.0501 | No |
| 190 | SLC12A2 |  |  | 8979 | -0.145 | 0.0447 | No |
| 191 | SQLE |  |  | 9209 | -0.194 | 0.0250 | No |
Table: GSEA details [plain text format]

  

Fig 2: HALLMARK\_G2M\_CHECKPOINT: Random ES distribution      
 Gene set null distribution of ES for **HALLMARK\_G2M\_CHECKPOINT**
